# Quality of life after total laryngectomy: impact of different vocal rehabilitation methods in a middle income country

**DOI:** 10.1186/s12955-020-1281-z

**Published:** 2020-04-03

**Authors:** F. G. R. Souza, I. C. Santos, A. Bergmann, L. C. S. Thuler, A. S. Freitas, E. Q. Freitas, F. L. Dias

**Affiliations:** 1 Researcher Psychologist from Department of Head and Neck Surgery, Brazilian National Cancer Institute, INCA, Praça da Cruz Vermelha, 23, Rio de Janeiro, 20230-130 Brazil; 2 Head and Neck Surgeon from the Department of Head and Neck Surgery, Brazilian National Cancer Institute, Rio de Janeiro, Brazil; 3 Clinical Research and Technology Incorporation Coordination, Brazilian National Cancer Institute, Rio de Janeiro, Brazil; 4 Speech-Language Pathologist from Department of Head and Neck Surgery, Brazilian National Cancer Institute, Rio de Janeiro, Brazil

**Keywords:** Health-related quality of life, Head and neck cancer, UW-QOL

## Abstract

**Introduction:**

The impact of advanced laryngeal cancer and its extensive surgical treatments cause significant morbidity for these patients. Total laryngectomy impacts essential functions such as breathing, communication and swallowing, and may influence the quality of life as well as affecting the social life of laryngeal cancer patients.

**Objective:**

Describe the quality of life and analyze the factors associated with the reduced quality of life in patients who have undergone total laryngectomy.

**Method:**

Observational cross-sectional study was carried out to evaluate the quality of life of patients who had undergone total laryngectomy due to laryngeal cancer. The fourth version of the UW-QOL Quality of Life Assessment Questionnaire from Washington University, validated for Portuguese, was used.

**Results:**

The study population was 95 patients, and the mean composite score of the QOL was 80.4. In the subjective domains the majority of the patients (38.9%) reported they felt much better at present compared to the month before being diagnosed with cancer. When questioned about how they evaluated their health-related quality of life, there was a predominance of those who considered it good (43.2%), and most considered they had a good quality of life (46.3%) considering personal well-being. The overall quality of life was considered good to excellent by 83.2% of the patients. Patients with tracheoesophageal prosthesis reported a better quality of life, compared to patients using an electrolarynx or esophageal voice.

**Conclusion:**

The high mean value of the composite score for quality of life revealed that the patients assessed their quality of life positively. The absence of vocal emission was the only variable associated with a lower quality of life within the composite score according to the UW-QOL questionnaire.

## Introduction

Laryngeal cancer accounts for approximately 25% of the malignant neoplasms in the head and neck region and 2% of all malignancies. Also it causes 83,000 deaths per year worldwide [1]. Estimates of around 6390 new cases of laryngeal cancer in men are expected in Brazil in the 2018–2019 period and 1280 new cases in women. The estimated risk for men is 6.17 cases per 100,000 and 1.20 per 100,000 for women [2].

The impact of advanced laryngeal cancer and its extensive surgical treatments cause significant morbidity for these patients. Total laryngectomy impacts essential functions such as breathing, communication and swallowing, and may influence the quality of life as well as affecting the social life of laryngeal cancer patients [3]. There are three different methods to carry out voice rehabilitation: esophageal voice, electrolarynx and a tracheoesophageal phonatory prosthesis. The rehabilitation of the voice using a tracheoesophageal prosthesis is currently considered the gold standard because it provides better vocal quality and longer phonation time than the other methods [4].

The World Health Organization (WHO) defines quality of life as “the individual’s perception of their position in life in the context of the culture and value systems in which they live, and in relation to their goals, expectations, standards and concerns.” [5] In research, the quality of life assessment tools are important tools to measure the effect of these health treatments on the lives of patients, as well as providing the feedback of patient experiences in a structured way [6].

Today, there are several specific instruments, which have been developed in other countries, available to assess the quality of life in patients with head and neck cancer. In this study, the fourth version of the University of Washington Quality of Life Questionnaire (UW-QOL) was used. This questionnaire provides a simple measure of health-related quality of life, and can be used for head and neck cancer patients. In clinical use it provides data concerning patients’ perceptions of the different types of treatments and can identify patients who are worse off and would benefit from a more appropriate intervention [7, 8].

In addition to having three general questions about their overall health-related quality of life, it is the only one with an open question for patients to comment on. It is considered a concise instrument, easy to understand and quick to apply [8].

There are also other questionnaires related to head and neck functions that are frequently used. FACT-HN (version 4.0) is a multidimensional and self-administered questionnaire with 5 domains: physical, social, familial, emotional, functional, and 12 questions specifically related to head and neck cancer. The instrument is concise and easy to apply and is sensitive to the evaluation of cancer patients in the acute and late phases of treatment; and also includes questions that are non-specific to the disease and treatment [9].

The QLQ-H&N35, which was developed by the European Organization for Research and Treatment of Cancer (EORTC), is a questionnaire widely used to evaluate the quality of life of patients with head and neck cancer. The EORTC QLQ-H&N35 should be applied together with the EORTC QLQ-C30 questionnaire, which evaluates the global quality of life in cancer patients in general; thus the two questionnaires appraise both global and specific domains in these patients. This questionnaire is considered to be sensitive to changes in the clinical staging of the patients; however it is much longer than the other QOL questionnaires for such patients [10].

The health-related quality of life of patients treated with total laryngectomy tends to decrease during treatment and stabilizes at around 12 months after surgery. These patients usually have a good long-term health-related quality of life following treatment despite the fact that total laryngectomy has a permanent and significant impacts on swallowing and speech [3].

This article aims to describe the quality of life and analyze the factors associated with the reduced quality of life in patients who have undergone total laryngectomy.

## Material and methods

In this work an observational cross-sectional study was carried out to evaluate the quality of life of patients who had undergone total laryngectomy due to laryngeal cancer.

The patients included in this study were those who were registered at the Brazilian National Cancer Institute from 2004 to 2012, with a confirmed histology of squamous cell carcinoma (SCC) of the larynx, in stages III and IV, with or without extension to the hypopharynx, who had undergone total laryngectomy and neck dissection, with or without adjuvant radiotherapy. The following individuals were excluded: patients under 18 years old; those hospitalized in the period of data collection; individuals who could not be contacted by telephone; those who did not attend the outpatient appointments; patients with the disease still active; and those who had undergone surgical treatment less than 6 months previously. Eligible patients who signed a Free and Informed Consent Term were interviewed consecutively from December 2009 to January 2013. The study was approved by the Ethics and Research Committee of the National Cancer Institute under number 96/09.

The fourth version of the UW-QOL Quality of Life Assessment Questionnaire from Washington University, validated for Brazilians, was used [11]. It has twelve question domains related to specific head and neck functions as well as those related to activity, recreation, pain, mood and anxiety. Each domain has three to five response options with scores ranging from 0 (worst) to 100 (best) that can be appraised individually or by the total score (composed of the mean of the twelve domains). There are also three subjective questions that do not have specific scores, and refer to comparisons between patients or groups of patients [11].

This research was carried out following the guidelines of Weymuller et al. for studies performed at a single institution [6]. The data collected included descriptive and independent variables related to socio-demographic characteristics (age, gender, race, marital status, educational level), clinical status (clinical staging TNM, 2017; topography of the ICD-O tumor), and treatments (type of surgery, radiotherapy, chemotherapy, and speech and language rehabilitation) [12].

A descriptive study of the study population was carried out, using the means and standard deviation for the continuous variables and frequency distribution for the categorical variables. The Kolmogorov-Smirnov test was used to evaluate the normal distribution of the quality of life scores and the independent quantitative variables. A dispersion diagram was performed to evaluate the linearity between the outcome and the independent quantitative variables. As the quantitative independent variables did not present a normal distribution, they were categorized according to a theoretical reference [13].

To evaluate the association between the independent variables and the quality of life domain scores, the differences between the means of each score were calculated, and then the statistical difference was obtained by analysis of variance with ANOVA and nonparametric Tukey tests. A 5% level of significance was used.

All analyzes were carried out using the SPSS 21.0 program. (IBM, São Paulo).

## Results

The results were based on a total of 95 laryngeal cancer patients with a mean age of 57.7 years old (± 9.0), a mean time after surgery of 47.5 months (± 42.2), and a mean UW-QOL composite score of 80.4.

The study population was predominantly male (90.5%), with a low educational level (51.6%), white (65.3%), and at the time of the interview lived with a partner (70.5%), presented clinical staging IV (64.2%) and had undergone radiotherapy (87.4%). The majority of the patients had undergone total laryngectomy and cervical emptying (92.6%), predominantly rehabilitated with a tracheoesophageal prosthesis (43.2%), followed by electrolarynx (33.7%), and at the interview with vocal emission (85.3%) (Table 1).

**Table 1:** Demographic and Clinical Characteristics of the Patients Cohort (*n* = 95)

Variable	No (%)
Sex	
Male	86 (90,5)
Female	9 (9,5)
Education, y	
1–7	49 (51,6)
≥ 8	42 (44,2)
No information	4 (4,2)
Ethnic Group	
White (caucasian)	62 (65,3)
Others	29 (30,5)
No information	4 (4,2)
Age, y	
≤ 60	56 (59,6)
> 60	38 (40,4)
67 (70,5)	
24 (25,3)	
4 (4,2)	
Marital Status	
Married	
Single	
No information	
Time since Total Laryngectomy, y	
≤ 2	31 (32,6)
> 2	64 (67,4)
T Stage	
III	34 (35,8)
IV	61 (64,2)
Adjuvant Treatment	
No	4 (4,2)
Radiotherapy	83 (87,4)
Radiotherapy + Chemotherapy	8 (8,4)
Tumor Site	
Larynx	91 (95,8)
Larynx and Hypopharynx	4 (4,2)
Surgery	
Total Laryngectomy + Neck Dissection	88 (92,6)
Total Laryngectomy + Neck Dissection + Pharyngectomy	7 (7,4)
Speech Therapy	
Esophageal Speech	22 (23,2)
Artificial Larynx	32 (33,7)
Tracheoesophageal Speech	41 (43,2)
Voice Emission	
No	14 (14,7)
Yes	81 (85,3)

Table 2 shows the results of the objective domains of UW-QOL. The mean composite score of the QOL was 80.4. In the subjective domains the majority of the patients (38.9%) reported they felt much better at present compared to the month before being diagnosed with cancer. When questioned about how they evaluated their health-related quality of life, there was a predominance of those who considered it good (43.2%), and most considered they had a good quality of life (46.3%) considering personal well-being. The overall quality of life was considered good to excellent by 83.2% of the patients (Table 3).

**Table 2:** Scores for the University of Washington Quality of Life Questionnaire (*n* = 95)

UW-QOLv4 Domain	Categories	*n* (%)
Pain	I have severe pain, not controlled by medication	0 (0,0)
	I have severe pain controlled only by prescription medicine	0 (0,0)
	I have moderate pain - requires regular medication	16 (16,8)
	There is mild pain not needing medication	13 (13,7)
	I have no pain	66 (69,5)
Appearance	I cannot be with people due to my appearance	1 (1,1)
	I feel significantly disfigured and limit my activities due to my appearance	0 (0,0)
	My appearance bothers me but I remain active	11 (11,6)
	The change in my appearance is minor	49 (51,6)
	There is no change in my appearance	34 (35,8)
Activity	I am usually in bed or chair and don’t leave home	0 (0,0)
	I don’t go out because I don’t have the strength	0 (0,0)
	I am often tired and have slowed down my activities although I still get out	13 (13,7)
	There are times when I can’t keep up my old pace, but not often	40 (42,1)
	I am as active as I have ever been.	42 (44,2)
Recreation	I can’t do anything enjoyable	0 (0,0)
	There are severe limitations to what I can do, mostly I stay at home and watch TV	6 (6,3)
	There are many times when I wish I could get out more, but I’m not up to it	10 (10,5)
	There are a few things I can’t do but I still get out and enjoy life	35 (36,8)
	There are no limitations to recreation at home or away from home	44 (46,3)
Swallowing	I cannot swallow because it “goes down the wrong way” and chokes me	2 (2,1)
	I can only swallow liquid food	3 (3,2)
	I cannot swallow certain solid foods	40 (42,1)
	I can swallow as well as ever	50 (52,6)
Chewing	I cannot even chew soft solids	2 (2,1)
	I can eat soft solids but cannot chew some foods	25 (26,3)
	I can chew as well as ever	68 (71,6)
Speech	I cannot be understood	0 (0)
	Only my family and friends can understand me.	27 (28,4)
	I have difficulty saying some words but I can be understood over the phone	58 (61,1)
	My speech is the same as always	10 (10,5)
Shoulder	I cannot work or do my hobbies due to problems with my shoulder	1 (1,1)
	Pain or weakness in my shoulder has caused me to change my work / hobbies	9 (9,5)
	My shoulder is stiff but it has not affected my activity or strength	23 (24,2)
	I have no problem with my shoulder	62 (65,3)
Taste	I cannot taste any foods	2 (2,1)
	I can taste some foods	14 (14,7)
	I can taste most foods normally	22 (23,2)
	I can taste food normally	57 (60,0)
Saliva	I have no saliva	1 (1,1)
	I have too little saliva	17 (17,9)
	I can taste most foods normally	30 (31,6)
	I can taste food normally	47 (49,5)
Mood	I am extremely depressed about my cancer	0 (0,0)
	I am somewhat depressed about my cancer	9 (9,5)
	I am neither in a good mood nor depressed about my cancer	10 (10,5)
	My mood is generally good and only occasionally affected by my cancer	26 (27,4)
	My mood is excellent and unaffected by my cancer	50 (52,6)
Anxiety	I am very anxious about my cancer	2 (2,1)
	I am anxious about my cancer	5 (5,3)
	I am a little anxious about my cancer	25 (26,3)
	I am not anxious about my cancer	63 (66,3)

**Table 3:** Patients Classification of Global Quality of Life (QOL)

UW-QOLv4 Global Questions	Categories	n (%)
Compared to the month before you developed cancer, how would you rate your health-related quality of life?	Much better	37 (38,9)
	Somewhat better	22 (23,2)
	About the same	27 (28,4)
	Somewhat worse	7 (7,4)
	Much worse	2 (2,1)
In general, would you say your health-related quality of life during the past 7 days has been:	Outstanding	18 (18,9)
	Very good	16 (16,8)
	Good	41 (43,2)
	Fair	20 (21,1)
	Poor	0 (0,0)
	Very poor	0 (0,0)
Overall quality of life includes not only physical and mental health, but also many other factors, such as family, friends, spirituality, or personal leisure activities that are important to your enjoyment of life. Considering everything in your life that contributes to your personal well-being, rate your overall quality of life during the past 7 days.	Outstanding	20 (21,1)
	Very good	15 (15,8)
	Good	44 (46,3)
	Fair	16 (16,8)
	Poor	0 (0,0)
	Very poor	0 (0,0)

The mean scores of the domains in the UW-QOL questionnaire for the clinical and demographic variables of the patients who had suffered advanced laryngeal malignancies are described in Table 4. Clinically the men presented better scores in most domains than the women and there was a statistically significant difference in the humor domain (*p* = 0.003). Those who lived without a partner reported a better clinical and statistical score for the activity domain (*p* = 0.033).

**Table 4:** Mean Scores for University of Washington Quality of Life Questionnaire Domains (*n* = 95)

Variables	Pain	*p* value	Appearance	*p* value	Activity	p value	Recreation	*p* value	Swallowing	*p* value	Chewing	*p* value	Speech	*p* value	Shoulder	*p* value	Taste	*p* value	Saliva	*P* value	Mood	*P* value	Anxiety	*P* value	Composite Score	*P* value
Sex																										
Male	88,08	0,905	80,81	0,365	82,56	0,900	**81,69**	0,222	82,30	0,571	84,50	0,621	60,95	0,833	**85,71**	0,164	80,27	0,903	76,40	0,874	**83,14**	0,003*	**86,51**	0,283	80,83	0,295
Female	88,89		75,00		83,33		**72,22**		77,89		88,89		59,44		**74,11**		81,44		77,89		**58,33**		**77,78**		76,33	
Age, y																										
≤ 60	86,59	0,491	**76,22**	0,059	82,32	0,880	81,71	0,725	82,22	0,898	83,34	0,598	64,32	0,142	81,37	0,248	81,34	0,767	**71,61**	0,117	81,10	0,916	83,00	0,327	79,42	
> 60	89,35		**83,33**		82,87		80,09		81,63		86,11		58,15		87,07		79,65		**80,28**		80,56		87,72		81,15	
																									0,498	
Education,y																										
1–7	89,80	0,563	80,10	0,829	82,65	0,725	78,57	0,252	**78,35**	0,077	83,67	0,648	60,71	0,773	83,04	0,477	80,98	1000	76,22	0,995	78,57	0,498	85,08	0,883	79,67	0,430
≥ 8	87,50		80,95		83,93		83,93		**86,64**		86,12		61,95		86,57		80,98		76,26		82,14		85,81		81,68	
Ethnic Group																										
White (caucasian)	90,73	AM	81,05	AM	84,68	0,244	**83,47**	0,128	81,32	0,597	85,76	0,601	63,03	0,230	83,94	0,665	82,27	0,511	75,82	0,827	82,26	0,255	85,55	0,938	81,52	0,289
Others	84,48		79,31		80,17		**75,86**		82,18		82,76		57,55		86,24		78,21		77,14		75,86		85,14		78,63	
Marital Status																										
Married	87,69	0,375	80,97	0,685	**80,97**	0,033*	81,34	0,831	81,72	0,745	83,09	0,283	59,82	0,251	84,63	0,977	**78,64**	0,174	**73,67**	0,123	78,73	0,343	84,64	0,600	79,42	0,120
Single	91,67		79,17		**89,58**		80,21		83,46		89,58		65,38		84,79		**87,50**		**83,42**		84,38		87,58		83,89	
T Stage																										
III	89,71	0,561	80,88	0,806	82,42	0,573	79,41	0,651	**86,38**	0,139	88,24	0,340	63,82	0,281	87,26	0,419	83,38	0,427	**81,41**	0,184	83,09	0,499	88,29	0,414	82,50	0,214
IV	87,30		79,92		87,50		81,56		**79,38**		83,07		59,13		83,13		78,70		**73,82**		79,51		84,23		79,24	
Tumor Site																										
Larynx	**88,74**	0,163	**79,67**	0,131	82,35	0,909	**80,22**	0,231	81,81	0,882	84,80	0,835	60,55	0,551	**85,40**	0,125	80,62	0,690	**76,23**	0,596	**80,49**	0,580	85,42	0,594	80,30	0,705
Larynx and Hypopharynx	**75,00**		**93,75**		82,79		**93,75**		83,50		87,50		66,75		**66,75**		75,00		**83,50**		**87,50**		91,75		82,68	
Time since TL, y																										
≤ 2	84,48	0,349	78,23	0,450	78,23	0,088	78,23	0,433	84,00	0,519	81,19	0,318	52,71	0,006*	80,74	0,271	78,55	0,652	74,26	0,564	78,23	0,482	**79,68**	0,078	77,21	0,076
> 2	89,45		81,25		84,77		82,03		80,86		86,72		64,73		86,48		81,27		77,64		82,03		**88,59**		81,95	
Surgery																										
TL + ND	88,35	0,729	**79,55**	0,174	82,39	0,631	**79,26**	0,016*	81,94	0,927	84,85	0,931	59,95	0,145	**83,76**	0,218	79,95	0,594	**75,42**	0,148	80,40	0,585	**84,92**	0,256	**79,83**	0,104
TL + ND + PH	85,71		**89,29**		85,71		**100,0**		81,14		85,71		71,57		**95,29**		85,71		**90,57**		85,71		**95,29**		**87,64**	
Radiation																										
No	93,75	0,555	75,00	0,557	81,25	0,873	75,00	0,594	**91,75**	0,364	87,50	0,835	66,75	0,551	**100,0**	0,187	**91,75**	0,398	**100,0**	0,072	**62,50**	0,129	**75,00**	0,348	83,35	0,624
Yes	87,91		80,49		82,69		81,04		**81,45**		84,80		60,55		**83,93**		**79,88**		**75,51**		**81.59**		**86,15**		80,28	
Speech Therapy																										
Esophageal Speech	85,23	0,722	**75,00**	0,296	**85,23**	0,203	**85,23**	0,256	**77,41**	0,456	**87,14**	0,166	45,32	< 0,001*	**89,41**	0,256	**74,23**	0,421	**74,27**	0,180	79,55	0,943	**75,82**	0,068	77,41	0,081
Artificial Larynx	89,06		**81,25**		**78,13**		**75,78**		**81,34**		**78,12**		56,38		**79,22**		**80,25**		**70,87**		80,47		**89,69**		78,38	
Tracheoesophageal Speech	89,02		**82,32**		**84,76**		**82,32**		**84,71**		**89,02**		72,59		**86,24**		**83,78**		**82,17**		80,71		**87,85**		83,60	
Voice Emission																										
No	83,93	0,376	**73,21**	0,117	87,50	0,262	78,57	0,685	**69,21**	0,019	**76,21**	0,162	**35,43**	< 0,001*	90,57	0,311	**66,64**	0,041	71,50	0,446	**73,21**	0,214	**69,14**	0,003*	**72,32**	0,007*
Yes	88,89		**81,48**		81,79		81,17		**84,07**		**86,42**		**65,20**		83,58		**82,75**		77,41		**82,10**		**88,45**		**81,80**	

*TL* Total Laryngectomy, *ND* Neck Dissection, *PH* Pharyngectomy

Values with statistical significance *p* < 0,05; Values in bold represent clinical significance

Patients who had undergone surgery more than 2 years before the date of the interview presented better quality of life scores with statistical significance for the speech domain (*p* = 0.006). Comparing those who had undergone total laryngectomy and neck dissection with or without pharyngectomy, those who performed the more extensive surgery had a better score. There was a statistical significance regarding speech and language rehabilitation in the speech domain (*p* ≤ 0.001): the patients with tracheoesophageal prosthesis reported a better quality of life, compared to patients using an electrolarynx or esophageal voice.

In the voice domain, there was a statistical significance in the swallowing (*p* = 0.019), speech (*p* ≤ 0.001), taste (*p* = 0.041), and anxiety (*p* = 0.003) domains and in the composite score (*p* = 0.007) for the individuals with vocal emission who described how their quality of life had improved. There was a difference of 9.48 points in the composite score between the patients with vocal emission (81.80) and without vocal emission (72.32).

## Discussion

This was the first quality of life study on patients who had undergone total laryngectomy, evaluated by the UW-QOL according to the speech and language rehabilitation center at the Brazilian National Cancer Institute. Advanced laryngeal malignancies and their extensive surgical treatments can result in various dysfunctions, with negative repercussions on the quality of life of such patients. A total of 95 patients with laryngeal cancer who had undergone total laryngectomy were included in this study. The results show that, in relation to the demographic and clinical characteristics, the study population was predominantly male (8 men for each woman), less than 60 years old, white, of low educational level, and at the time of the interview lived with a companion, had clinical staging IV, and had undergone radiotherapy. Similar results have been obtained in other studies where the patients with laryngeal cancer were mostly men and with low educational level [3, 4, 14].

The replies to the questions concerning the subjective domains of the questionnaire showed that 78.9% of the patients considered their quality of life to be good to excellent and 90.5% indicated that their health was equal to or better than before diagnosis. These results were better than those reported in the population studied by Vartanian et al. where 59.3% of patients considered their quality of life to be good to excellent, and 74.0% indicated that their health was equal to or better than before surgery [11].

The female participants had a lower score in the mood domain, showing a greater chance of developing depression after treatment. These data were confirmed by the studies of Rogers et al. and Silveira et al. indicating that women suffer greater negative impacts on their quality of life than men [15, 16].

Although the health-related quality of life of patients treated with total laryngectomy tends to decline during treatment, it stabilizes at around 12 months post-surgery [3]. In a recent study on the importance of UW-QOL domains for head and neck cancer patients, Metcalfe et al. demonstrated that in the first 12 months after treatment there is a minor oscillation of items that patients consider important. However, after this period, patients attach greater importance to the swallowing, chewing and speaking domains, a tendency that continues along their life time [17].

In the study conducted by Eadie and Bowker [18], higher quality of life scores were associated with post-surgical times greater than 2 years. This was mainly for the speech domain, which is in agreement with the observations by Metcalfe et al. [17] and may be related to the possibility of better speech and language rehabilitation methods nowadays. Also, those who had more effective vocal emission at the interview were less anxious and evaluated their quality of life better in relation to their swallowing and speech than others.

After total laryngectomy, patients need to learn a new form of oral communication and how to deal with changes in breathing and swallowing. Although the literature shows that a large part of these patients have managed to overcome these challenges in 12 months after total laryngectomy, there are still some individuals who suffered a significant impact on their quality of life in the long term [3, 16, 17]. Quality of life questionnaires are focused on the dysfunctions resulting from the treatments over a short period of time. They do not contemplate the adaptation and cognitive coping that occurs over a longer time period, which may lead to an incongruity between the dysfunctions observed in the patients and the meaning in their lives [19].

Compared to the electrolarynx and the esophageal voice, patients who used the tracheoesophageal prosthesis had significantly better UW-QOL scores in speech. These results suggest that the tracheoesophageal prosthesis can be considered the best method of speech and language rehabilitation, resulting in a better quality of life and better vocal satisfaction. These data are in agreement with Oozeer et al. [3] and Balm et al. [20], who affirm that the restoration of the voice through the tracheoesophageal prosthesis offers the best possibility of oral communication for patients who have undergone total laryngectomy, and should be considered the gold standard for vocal rehabilitation. The preference for the vocal prosthesis also lies in the fact that this device can be implanted at the time of total laryngectomy [21].

In the present study the type of surgery did not influence the appearance domain, although a negative impact was expected for patients undergoing extensive treatments because of the liability to incur major cosmetic defects as well as physical and functional sequelae. Gill et al. conducted a study comparing groups of patients with head and neck cancer, their caregivers, and health care staff about their concerns and the most important aspects related to treatment. The appearance was considered a factor of great importance only for the health care group, showing that, in agreement with the results presented here, for the patients or their companions the concern with appearance was not so important [22].

Major et al. performed a study with 24 patients comparing who underwent total laryngectomy followed by radiation therapy and patients who received concomitant chemotherapy and radiation therapy [23]. The study showed that patients who did received surgery were more limited in their activities of daily living than the other group. In the same aspect, a study conduced by Guibert et al. evidenced that is a little difference in quality of life betweenpatients with total laryngectomy or organ conservation [24]. Unlike these studies, ours did not compare non-surgical with surgical patients. The focus of our study was the evaluation of quality of life according to the different methods of vocal rehabilitation in total laryngectomized patients, with time interval up to interviews over 2 years, similar to the aforementioned studies.

The only variable that influenced the quality of life composite score was the voice. The mean composite score of patients without vocal emission was 9 points less in relation to those with vocal emission. A study by Eadie and Bowker in 2012 demonstrated that the use of the traditional variables from the literature is not sufficient to establish associations with the quality of life domains [18].

The present study demonstrated that the UW-QOL questionnaire is an important evaluation tool and its incorporation into clinical practice is of great relevance because it can help to improve and measure the effectiveness of the treatments and their sequels. The quality of life of these patients can be improved through interventions that support the impact of the disease and its treatments [25, 26]. In this work we were able to compare the three groups of vocal rehabilitation through the questionnaire and to prove the superiority of the tracheoesophageal prosthesis in our population, justifying the investment of tracheoesophageal prosthesis, which only the Brazilian National Cancer Institute provides for free.

Other public hospitals in Brazil have limited access to this method, and because it is the hospital of reference for cancer treatment, it is important that Brazilian National Cancer Institute reaffirm that this method of vocal rehabilitation is superior and necessary for the better quality of life of total laryngectomized patients.

The importance of incorporating quality of life into daily practice and the need for a multidisciplinary team trained and coherent in the oncological treatment aimed at the integral care of the patients should be emphasized [25]. Prior identification of concerns, depression and anxiety in patients with cancer of head and neck is of great importance because depression is underdiagnosed in cancer patients [26, 27]. Another factor that could influence a patient’s quality of life is the fear of a relapse, characterized by the fear of the cancer returning, which according to Ghazali et al. is present in 35% of patients who have survived cancer [28].

This study had some strengths, since it focused on one of the main tumor sites in the head and neck area, and the patients were grouped according to the different methods of speech and language rehabilitation methods that are carried out at the Brazilian National Cancer Institute (INCA).

However, in order to minimize the information and selection biases, the data collection was performed by a single researcher and the scores were measured by an evaluator trained to use this QOL instrument, but who was not part of the research team. Furthermore due to the low incidence of this treatment and its pathology in Brazil, the patients chosen to participate were those who had undergone the surgical procedure before the beginning of the study (December 2009). One limitation of this study may be related to recall bias, since the patients included had undergone the surgical procedure before the beginning of the study (december 2009), and in majority were interviewed more than 2 years after the procedure. This can also be connected with cognitive bias, once most of the patients were younger than 60 years, with the long-term therapy and rehabilitation, this patients may refer a better QOL due to feel more greatful to be alive despite the functional and aesthetic difficulties.

A limitation due to the cross-sectional design used is that the patients included in the study may not be representative of the total population of the patients who underwent treatment at NCI, but those who survived it. Another possible limitation is the fact that quality of life was measured after treatment and these patients could have already presented a loss of QOL at the time they were diagnosed with cancer.

As in the present study, few studies have examined the psychosocial variables and their relationships with quality of life, such as coping, which has shown to be an important association with quality of life in the literature. Coping strategies are cognitive and behavioral efforts used to deal with internal and external demands of stressful circumstances. Individuals use different coping patterns in different circumstances [29].

Despite the methodological limitations inherent to the design, the results were able to describe the general aspects of the quality of life in this population of the Institute. These results can be used in the planning and evaluation of actions for patients undergoing treatment for head and neck cancer.

## Conclusions

The population included in the study was mostly composed of men of low educational level with clinical staging IV, who had undergone total laryngectomy with cervical emptying, adjuvant radiotherapy and predominantly rehabilitated with tracheoesophageal prosthesis. At the time of the interview, vocal emission was observed in most patients. There was a significant improvement in quality of life after treatment, and the majority of the patients considered the quality of life at the time of the interview good to excellent.

The results showed that the worst quality of life scores for patients who had undergone total laryngectomy were in the domains for mood, activity, rehabilitation with the esophageal voice and lack of vocal emission. The high mean value of the composite score for quality of life revealed that the patients assessed their quality of life positively. The absence of vocal emission was the only variable associated with a lower quality of life within the composite score according to the UW-QOL questionnaire.

## Data Availability

Please contact author for data requests.

## Abbreviations

IBM: International Business Machines Corporation
ICD-O: International Classification of Diseases for Oncology
QOL: Quality of Life
SCC: Squamous cell carcinoma
SPSS: Statistical Product and Service Solutions
UICC TNM classification: The UICC TNM Classification is an anatomically based system that records the primary and regional nodal extent of the tumor and the absence or presence of metastases. Each individual aspect of TNM is termed as a category: T category describes the primary tumor site N category describes the regional lymph node involvement M category describes the presence or otherwise of distant metastatic spread
UW-QOL: University of Washington Quality of Life Questionnaire
WHO: World Health Organization
